# Bioinspired Spinosum Capacitive Pressure Sensor Based on CNT/PDMS Nanocomposites for Broad Range and High Sensitivity

**DOI:** 10.3390/nano12193265

**Published:** 2022-09-20

**Authors:** Yanhao Duan, Jian Wu, Shixue He, Benlong Su, Zhe Li, Youshan Wang

**Affiliations:** 1 National Key Laboratory of Science and Technology on Advanced Composites in Special Environments, Harbin Institute of Technology, Harbin 150090, China; 2 Center for Rubber Composite Materials and Structures, Harbin Institute of Technology, Weihai 264209, China

**Keywords:** flexible capacitive sensors, bioinspired spinosum, CNT/PDMS nanocomposite, tires

## Abstract

Flexible pressure sensors have garnered much attention recently owing to their prospective applications in fields such as structural health monitoring. Capacitive pressure sensors have been extensively researched due to their exceptional features, such as a simple structure, strong repeatability, minimal loss and temperature independence. Inspired by the skin epidermis, we report a high-sensitivity flexible capacitive pressure sensor with a broad detection range comprising a bioinspired spinosum dielectric layer. Using an abrasive paper template, the bioinspired spinosum was fabricated using carbon nanotube/polydimethylsiloxane (CNT/PDMS) composites. It was observed that nanocomposites comprising 1 wt% CNTs had excellent sensing properties. These capacitive pressure sensors allowed them to function at a wider pressure range (~500 kPa) while maintaining sensitivity (0.25 kPa^−1^) in the range of 0–50 kPa, a quick response time of approximately 20 ms and a high stability even after 10,000 loading–unloading cycles. Finally, a capacitive pressure sensor array was created to detect the deformation of tires, which provides a fresh approach to achieving intelligent tires.

## 1. Introduction

Flexible pressure sensors have become a rapidly growing research area with significant progress in 5G communication and artificial intelligence [1]. Currently, flexible pressure sensors are widely used in the monitoring field, such as in examining human health [2,3] and structural health detection [4,5]. Compared with rigid sensors, flexible pressure sensors can withstand various deformations, such as bend, compression and torsion [6,7]. With the introduction of modern technologies, such as network technology and cloud computing, intelligence has become a significant trend in automotive technology development and tires intelligence. The tires are the only part that contacts the vehicle and the road and have attracted considerable attention in improving vehicle safety and reducing fuel consumption. Measuring or estimating tire force is vital for analyzing and controlling vehicle behavior [8]. The contact patch features and the contact pressure of the tires are significant for the force estimation [9]. However, measuring exact forces in the real world has become a concern for researchers. The sensors built into the tires to form intelligent tires can provide reliable quantities of tire variables compared with traditional test methods based on onboard sensors [10]. The use of flexible pressure sensors for tires is a promising method for detecting the contact pressure of tires. Matsuzaki et al. fabricated a novel rubber-based sensor and attached it to the inner surface of a tire. The experiment showed that the sensor could accurately monitor the tire’s behavior [11]. Son et al. examined a reliable triboelectric bicycle tire by inserting a dielectric electrode layer between the tire tread and the inner tube [12]. The pressure and deformation of the tire can be monitored in real time by the sensor. According to working mechanisms, flexible pressure sensors are mainly based on piezoresistive [13,14], capacitive [15,16], piezoelectric [17,18] and triboelectric effects [19,20]. Capacitive pressure sensors have attracted considerable research attention owing to their simple structure, good repeatability, low loss and temperature independence, which are considered the ideal choice for structural health testing [21]. These pressure sensors adopt a sandwich structure consisting of two electrode layers at the top and bottom and one sensing layer in the middle. Polydimethylsiloxane (PDMS) has been employed widely as a flexible substrate because of its excellent characteristics, including flexibility, biocompatibility and mechanical properties [22]. With the progress of materials science, materials with different microstructures have been used extensively to fabricate pressure sensors. In particular, carbon-based functional materials, such as nanotubes [23], graphene [24], carbon black [25] and graphene oxide [26], have been used broadly as conductive fillers in sensors because of their low cost and outstanding electrical and mechanical properties. Adding conductive fillers to the dielectric layer can produce a higher dielectric constant and improve the signal magnitude. Furthermore, to enhance the sensitivity of capacitive sensors, the compressibility and effective dielectric constant change value can be improved by patterning the dielectric layer, such as by adding microstructures [27,28]. In 2010, Mannsfeld et al. first added a micropyramid structure to capacitive sensors, significantly improving their sensitivity [29]. In addition to the micropyramid structure, some other microstructures, such as micropillar [30,31], microdome [32,33], wrinkle [34] and microporous [35,36], have been explored. Despite the considerable progress on capacitive sensors, the large-scale preparation of low-cost, high-sensitivity and broad-range capacitive pressure sensors presents enormous challenges. Many structures in nature provide humans with examples of good design, which have inspired researchers in bioinspired pressure sensors [37]. Skin plays an important role in the perception of force, and the dermis with a spinosum microstructure makes skin sensitive to external forces [38]. Bioinspired pressure sensors with a spinosum surface have been used for the development of low-cost pressure sensors with a high sensitivity and a large linearity [39]. Little research has been conducted on the spinosum microstructure for capacitive pressure sensor design. Flexible sensor arrays can achieve spatial pressure distribution monitoring and real-time trajectory mapping, which are used widely in various fields [40]. Using flexible sensor arrays on tires may achieve real-time detection of their contact pressure and provide more accurate quantities of the tire variables.

This article reports a bioinspired spinosum capacitive pressure sensor based on carbon nanotube/polydimethylsiloxane (CNT/PDMS) nanocomposites for detecting the contact pressure of tires. CNT/PDMS-based spinosum pressure sensors inspired by the epidermis tissue structure in human skin were fabricated. The spinosum microstructure of the dermis has a high similarity in topography with abrasive paper. A capacitive pressure sensor with a high sensitivity and a large sensing range can be achieved using abrasive papers with different surface roughness as a template. The flexible pressure sensor exhibited a high sensitivity of 0.25 kPa^−1^ in the range of 0–50 kPa, a wide effective working range of 0–500 kPa, ultrafast response and relaxation times of 20 ms and excellent cycling stability (>10,000 cycles). As shown in Table 1, our sensor exhibits a higher sensitivity and a fast response time compared with reported capacitive pressure sensors in the literature. CNT/PDMS-based spinosum pressure sensors are excellent candidates for health monitoring. Based on the novel design of our sensor, a sensor array was used successfully in the quantitative monitoring of contact pressure on a tire.

## 2. Materials and Methods

### 2.1. Preparation of Spinosum Microstructure CNT/PDMS Films as the Dielectric Layer

Commercial abrasive papers (Hubei Yuli Abrasive Belt Group, Hubei, China) with three roughness values, no. 80, 320 and 600, were utilized as templates. Nanocomposites with different compositions (mass ratio of PDMS/CNTs = 10:X, X = 0, 0.05, 0.1 and 0.15) were prepared to fabricate the composite dielectric layer. CNTs (purity: 95%, diameter: 3–15 nm, length: 15–30 μm, Shenzhen Suiheng Technology Co., Ltd., Guangdong, China) were dispersed in alcohol (10 g, 99.7%, Ziyansheng Fine Chemical Co., Ltd., Shanghai, China) for 2 h with ultrasonic assistance. Then, a PDMS base (9.1 g) (Sylgard 184, Dow Corning Co., Ltd., Midland, MI, United States) was dispersed in the alcohol/CNT solution for 2 h with magnetic stirring. Then, the alcohol was allowed to evaporate at 150 °C. After the solution cooled to room temperature (22 °C), a curing agent (0.9 g) (Sylgard 184, Dow Corning Co., Ltd., Midland, MI, United States) was added and the solution was stirred for 1 h. Then, the degassed CNT/PDMS solution was poured onto the abrasive paper and cured at room temperature for 12 h. Finally, the CNT/PDMS films were carefully peeled off to obtain the spinosum microstructure. The thickness of the dielectric layer was 1 mm.

### 2.2. Preparation of the Sensor Array

The bottom and top electrodes of the sensor array, both containing strip electrodes, were orthogonally mounted face to face with the CNT/PDMS spinosum dielectric layer, forming a sandwich structure. A capacitive pressure sensor array was then created.

### 2.3. Characterization of the Morphology and Performance of the CNT/PDMS-Based Spinosum Pressure Sensors

The morphology and structure of the fabricated sensors were characterized via a field emission scanning electron microscope (SEM, MERLIN, Zeiss, Jena, Germany). The fabricated sensors’ 3D morphology and structure were characterized via an optical microscope (DSX 510, Olympus, Tokyo, Japan). The contact area of the dielectric layer was characterized with a high-speed CCD camera (Yvision Technology Company, Guangdong, China). The loading of applied force was carried out with a testing machine (WDW-02, STAR Testing Technology Co., Ltd., Shandong, China), while the electrical signals of the pressure sensors and the permittivity were recorded at the same time via an LCR meter (TH2829A, Changzhou Tonghui Electronic Co. Ltd., Jiangsu, China) at a 100 kHz frequency with a 0–1 V alternating voltage bias. A fatigue-testing machine (FLPL203E, FULETEST Instrument Technology Co., Ltd., Shanghai, China) was used to test the response/release time, and the depressing speed and rising speed were both 1000 mm/min. The stability test also used the fatigue testing machine (FLPL203E, FULETEST, Instrument Technology Co., Ltd., Shanghai, China), and the depressing speed and rising speed were both 1000 mm/min.

### 2.4. Finite Element Analysis

Finite element analysis (FEA) was performed using the commercial package ABAQUS. The CNT/PDMS-based spinosum microstructure dielectric layer was modeled as an incompressible material with Young’s modulus E~1.4 MPa. The Cu electrode was simply treated as a rigid plate and compressed downward. All contact interactions were assumed to have friction with a friction coefficient of 0.15 without penetration.

## 3. Results and Discussion

### 3.1. Designs for Spinosum Capacitive Pressure Sensors

**Figure 1:**
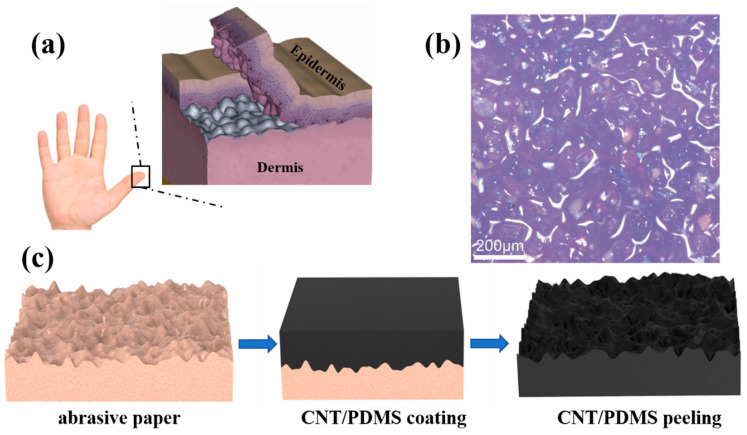
(**a**) Schematics of the microstructure of human epidermis. (**b**) Optical microscopy image of the no. 400 abrasive paper. (**c**) The fabrication process of the spinosum microstructure pressure sensor.

So far, nature has evolved to optimize structures to adapt to complicated environmental conditions, inspiring many engineers and scientists. Skin, one of the largest organs of the human body, can interact with the surrounding environment. Because of the spinosum of the dermis, the skin can sensitively sense stimulation from the environment (Figure 1a). The sensitivity and detection accuracy of pressure sensors can be improved through introducing the spinosum microstructures, which are attributed to the concentrated local stress at the contact region. As shown in Figure 1b, the surface of abrasive paper displays smoothly interconnected ridges and dispersive holes. Because the spinosum microstructure has a similar morphology to that of abrasive paper, we used abrasive papers as a temple to fabricate the spinosum microstructure. Figure 1c depicts the schematic illustration of the fabrication process of the spinosum pressure sensor. The spinosum pressure sensor was fabricated as follows: first, the prepared CNT/PDMS nanocomposite was coated on the abrasive paper template through a blading method and cured at room temperature. Second, the CNT/PDMS film was peeled off from the abrasive paper to obtain the spinosum microstructure. Third, the as-prepared spinosum microstructure CNT/PDMS films were cut into regular sizes (10 mm × 10 mm).. Subsequently, a piece of copper foil tape (Dongguan Xinshi Packaging Materials Co., Ltd. Guangdong, China) was attached to the side of the polyethylene (PE) substrate as bottom electrode. Then another piece of copper foil tape was attached to the flat side of the CNT/PDMS film as top electrode. The CNT/PDMS film was sandwiched between two copper electrodes. Finally, the copper wires were attached onto the copper foil to complete the sensor fabrication. The multilayer was packaged between two transparent and soft 3M tape. As we know, the traditional lithography template method to fabricate pressure sensors is expensive. Compared with the lithography template method, using abrasive paper as a template to fabricate pressure sensors is very cost-effective. The above strategy has the advantages of simplicity, economy and ease of operation, and the filling materials are not expensive.

### 3.2. The Performance of Spinosum Capacitive Pressure Sensors

**Figure 2:**
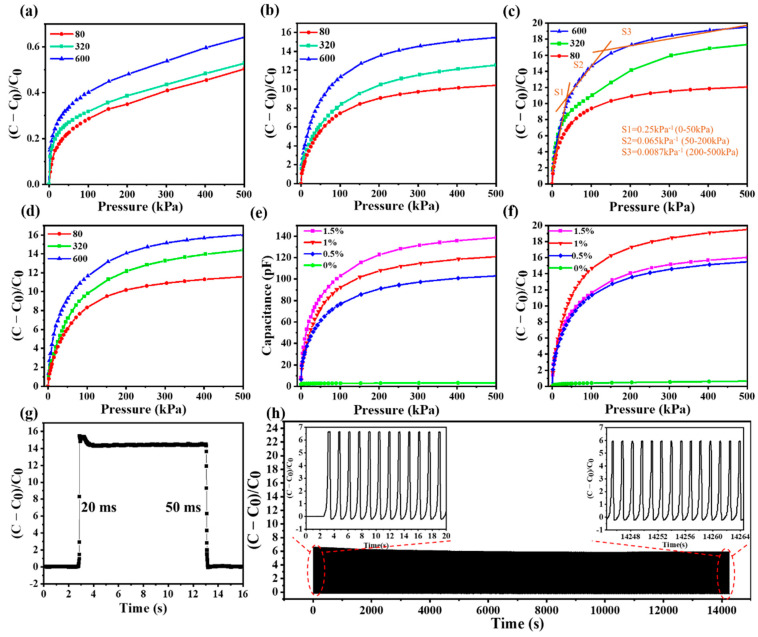
Sensing performances of the pressure sensors. (**a**) Pressure response of relative capacitance change in the sensor with (**a**) 0, (**b**) 0.5, (**c**) 1 and (**d**) 1.5 wt% CNT doping content using different abrasive papers. (**e**) Pressure response of the absolute capacitance of the sensor using abrasive paper no. 600 under different doping contents of CNTs. (**f**) Pressure response of the relative capacitance of the sensor using abrasive paper no. 600 under different doping contents of CNTs. (**g**) Response time and recovery time of the spinosum pressure sensor. (**h**) Durability test of the pressure sensor for 10,000 loading/unloading cycles at the pressure of 30 kPa.

The pressure sensitivity (S) of capacitive pressure sensors can be defined as S = (ΔC/C_0_)/ΔP, where ΔC is the relative change in capacitance, C_0_ represents the initial capacitance when no pressure is applied and ΔP is the change in applied pressure. To investigate the effect of the spinosum microstructure on capacitive sensors, the distributed spinosum microstructure PDMS with increasing roughness was fabricated via abrasive papers no. 80–600. The as-prepared spinosum PDMS films were cut into 10 mm × 10 mm sizes and sandwiched between two copper electrodes. In order to characterize the capacitive responses of the sensors to external pressures, a high-precision test system containing a motorized test stand connected with a force gauge, an LCR meter and computers was used. The pressure load on the pressure sensors was precisely controlled via the force gauge, and the capacitive signals were collected through the LCR meter. In Figure 2a, the ΔC/C_0_ versus pressure change of the PDMS-based spinosum pressure sensors with different degrees of roughness using abrasive paper templates no. 80–600 is shown. The result indicates that the spinosum pressure sensor using abrasive paper template no. 600 exhibits the highest sensitivity compared to sensors of other degrees of roughness. However, the sensitivity is relatively low. To further improve the performance of the spinous pressure sensor, the dielectric layer was doped with CNTs to increase the dielectric constant.

The influence of the CNT doping content and the roughness of the abrasive surface on the performance of spinosum microstructure sensors have been investigated in this study. By using abrasive papers no. 80–600 as templates, the spinosum pressure sensors with different CNT doping content (0.5, 1 and 1.5 wt%) were fabricated and tested the performance of the sensors via the test system.

The CNT/PDMS-based spinosum pressure sensor exhibits a high sensitivity and a wide detection range. As shown in Figure 2b–d, similarly to the previous result, the ΔC/C_0_ versus pressure change for different CNT doping content (0.5, 1 and 1.5 wt%), the CNT/PDMS-based spinosum pressure sensor shows a similar regular, which is that the sensor using abrasive paper template no. 600 exhibits the highest sensitivity. The capacitance changes of spinosum pressure sensors fabricated with abrasive paper no. 600 with different amounts of CNTs (0, 0.5, 1 and 1.5 wt%) are plotted in Figure 2e. The regular is obvious: the absolute capacitive of the CNT/PDMS-based spinosum pressure sensors increase with the doping content of CNTs. However, the regular is different when the capacitance change is normalized by initial capacitance. The CNT/PDMS-based spinosum pressure sensors with 1 wt% CNT doping content show the highest sensitivity among the samples tested (Figure 2f). The sensitivity of 1 wt% doping CNT/PDMS-based spinosum pressure sensors prepared via abrasive paper no. 600 is 0.25 kPa^−1^ in the low-pressure range of 0–50 kPa, 0.065 kPa^−1^ for 50–200 kPa, and 0.0087 kPa^−1^ in the high-pressure range of 200–500 kPa.

Furthermore, the optimized design parameters (1 wt% CNT doping and fabricated from the abrasive paper no. 600) were selected from the above experiments and used to study the response time and the stability of the sensor. To investigate the response speed, the response and relaxation time of the pressure sensor were estimated in the process of loading/unloading. As described in Figure 2g, a response time of 20 ms and a relaxation time of 50 ms were observed, which indicate its fast pressure response and potential application in the monitoring field. Moreover, as shown in Figure 2h, the pressure sensor exhibits robust durability in the process of loading/unloading a massive pressure of 30 kPa up to 10,000 cycles. As we can see in the insets of the magnified view, the capacitance signal amplitude did not decrease significantly throughout the 10,000 load/unload test cycles.

### 3.3. The Effect of the Mesh Number of Abrasive Papers and CNT Doping Content on the Sensing Property

**Figure 3:**
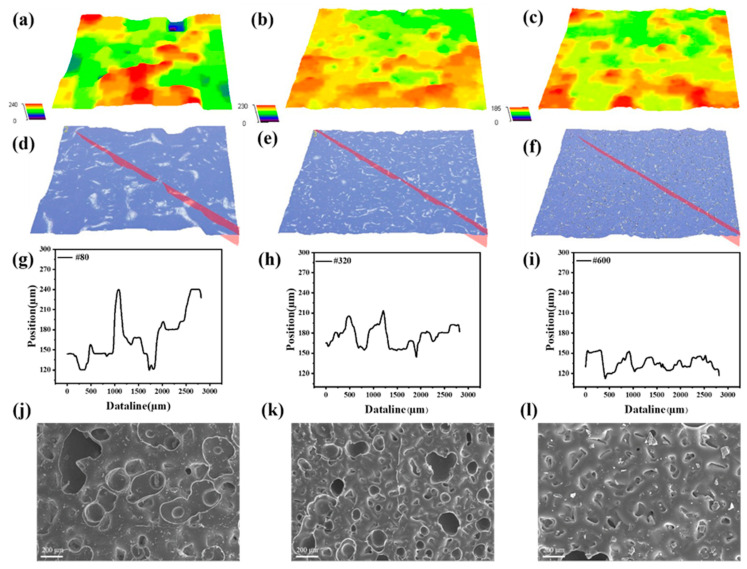
Characterization of the pressure sensors. The 3D morphology of spinosum CNT/PDMS dielectric layer using abrasive papers no. (**a**) 80, (**b**) 320 and (**c**) 600. The position of the marker line of the CNT/PDMS dielectric layer using abrasive papers no. (**d**) 80, (**e**) 320 and (**f**) 600. Height profile corresponding to the marked line on the diagonals using abrasive papers no. (**g**) 80, (**h**) 320 and (**i**) 600. The SEM images of the spinosum CNT/PDMS dielectric layer using abrasive papers no. (**j**) 80, (**k**) 320 and (**l**) 600.

Figure 3a–i show the typical optical image of a spinosum microstructure CNT/PDMS dielectric layer using abrasive papers no. 80, 320 and 600, indicating that the spinosum microstructure of the abrasive paper surface is well-replicated. Generally, the height of the spinosum microstructure decreases as the mesh number of the abrasive paper increases, and the density of the spinosum increases as the mesh number of the abrasive paper increases. The successful fabrication of CNT/PDMS-based spinosum dielectric layers was confirmed through the SEM image, as shown in Figure 3j–l. The microstructures are distributed all over the surface, and as the roughness of the abrasive paper decreases, the microstructures’ density increases, and the dimension of these microstructures decreases.

**Figure 4:**
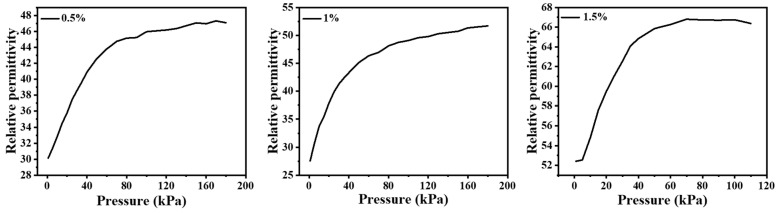
Relative permittivity of the PDMS/CNT nanocomposites with 0.5, 1, and 1.5 wt% CNT under different pressures.

Figure 4 shows that the relative permittivity of CNT/PDMS nanocomposites with different CNT doping content increases as the pressure increases. When the doping content of CNTs reaches 1 wt%, its relative permittivity changes most obviously. In fact, the relative permittivity increase can be explained according to the percolation theory: ε ∝ (ρ-ρ_0_)s, where ε is the relative permittivity, ρ is the percolation threshold and ρ_0_ is the CNT concentration [46]. The distance of CNTs decreases during the compression process, which leads to a decline in ρ and an increase in ε.

**Figure 5:**
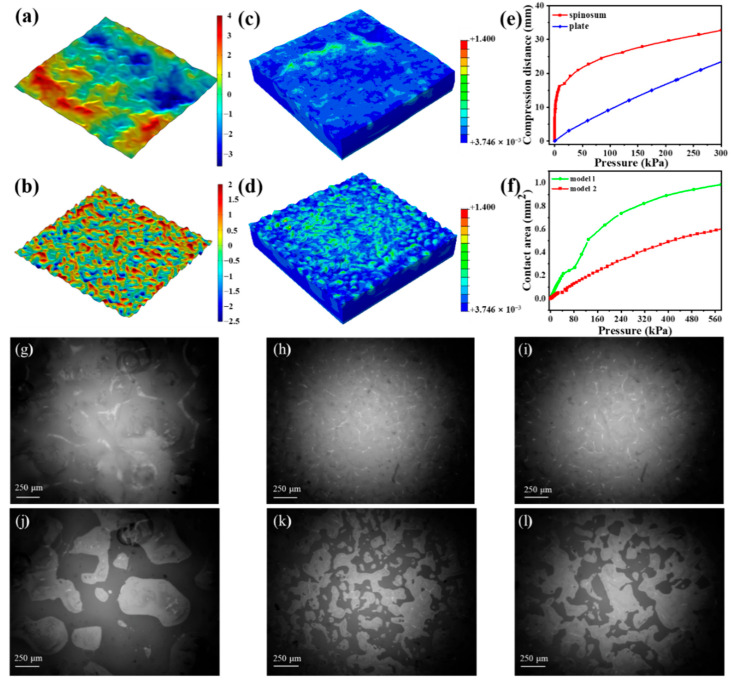
Sensing mechanisms of the pressure sensors. Schematic of the simulation (**a**) model 1 and (**b**) model 2 with different spinosum microstructures. FEA simulation showing the stress distribution of (**c**) model 1 and (**d**) model 2 at a pressure of 100 kPa. (**e**) The FEA simulation result of the compression distance variation for the spinosum and plate dielectric layer. (**f**) The FEA simulation result of the contact area variation between the dielectric layers with different architectures. The image of the spinosum dielectric layer using different abrasive papers no. (**g**) 80, (**h**) 320 and (**i**) 600 without pressure. The image of the contact area of the spinosum dielectric layer using different abrasive papers no. (**j**) 80, (**k**) 320 and (**l**) 600 at a pressure of 180 kPa.

To further reveal the response mechanism of the sensor, the force distribution and deformation process of the spinosum microstructure were studied through finite element analysis. Two models with different roughness values have been conducted to simulate the compression process of the sensor. Model 1 (Figure 5a) has a low density of spinosum, but the height of the spinosum is large, which can represent abrasive papers with a low mesh number (the surface height is represented by color). Model 2 (Figure 5b) has a high density of spinosum but the height of the spinosum is low, which is similar to the abrasive papers with a high mesh number. Figure 5c,d show the stress distribution after the loading of 100 kPa for model 1 and model 2, respectively. As we can see, when under pressure, the stress of the spinosum structure is concentrated at the contact peak. The spinosum microstructure with a lower roughness has a more homogeneous pressure distribution than that with a greater roughness, and the stress concentration area of the spinosum microstructure with a lower roughness is much larger than that with a greater roughness. Thus, the relative permittivity of the underlying dielectric layer will be dramatically increased, which contributes to the stress concentration of the spinosum microstructure. Because model 2’s stress concentration area is more extensive than model 1’s, the relative permittivity changed obviously. Thus, the CNT/PDMS-based spinosum pressure sensor using abrasive paper no. 600 has a better performance.

By introducing the spinosum microstructure, the dielectric layer can more readily deform with applied pressure and be more easily compressed than without the microstructure (Figure 5e). As shown in Figure 5f, when under the same pressure, the contact area with electrodes in model 2 is smaller than in model 1. The roughness will decrease as the mesh number of the abrasive paper template increases; the spinosum microstructure with an extensive range of height differences is easily deformed due to stress concentration. Thus, the contact area with electrodes in model 1 is larger than in model 2. The simulation results are consistent with the experimental results. A transparent glass sheet was utilized to replace the electrodes for easy observation, and a 1.8 kg weight was placed above the sensor. A high-speed CCD camera was used to characterize the contact area change in the spinosum dielectric layer. As depicted in Figure 5g–l, when under the same pressure, the contact area (shaded area) gradually becomes smaller as the number of the abrasive papers’ mesh increases. When under compression progress, the contact area of the spinosum dielectric layer gradually increases. The contact area of the spinosum microstructures with low roughness saturates quickly, contributing to a low sensitivity level.

In summary, the capacitance change in the pressure sensor is a synergistic effect of the spinosum microstructure and CNT doping. The spinosum microstructure leads to local stress amplification and the contact area change in the dielectric layer with electrodes. The spinosum microstructure can provide additional compressibility by accommodating compressed protrusions. Moreover, the viscoelasticity of the elastomer is reduced because of the microstructure, resulting in a faster sensor response. In addition, introducing the CNTs to the dielectric layer can significantly improve the capacitive magnitude because the dielectric constant is improved and the relative permittivity can change with the external pressure. The relative permittivity of the dielectric layer will be dramatically amplified by the spinosum microstructure. It is through the above-mentioned synergistic effect that the excellent performance of the sensor can be caused.

### 3.4. Application of Spinosum Capacitive Pressure Sensor

**Figure 6:**
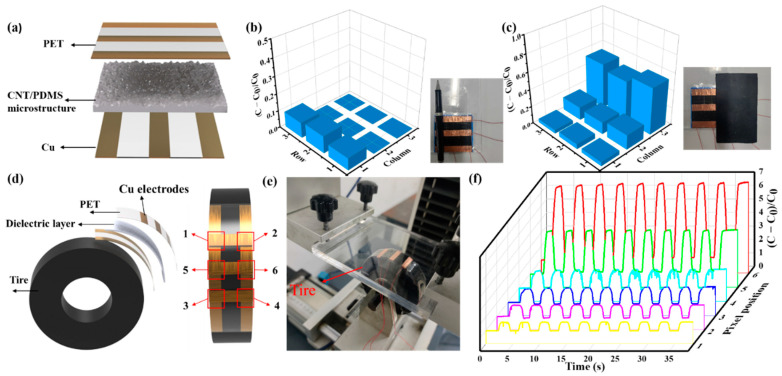
Application of the spinosum pressure sensors. (**a**) Schematic of the 3 × 3 pixelated pressure sensor array. Mapping of pressure distribution on the 3 × 3 sensor array upon (**b**) placing a pen and (**c**) placing a rectangular mass. (**d**) Schematic of the structure of the intelligent tire and the pixel position. (**e**) Photograph of the intelligent tire and test platform. (**f**) The capacitance signal at each pixel position of the intelligent tire under ten decompression cycles.

As shown in Figure 6a, a 3 × 3 sensor array has been fabricated to map pressure distribution, which consists of three strip copper foils as top and bottom electrodes and a 1 wt% CNT doping spinosum CNT/PDMS film using the abrasive paper template no. 600 as the dielectric layer. As shown in Figure 6b,c, this array can clearly distinguish the position and weight of different things, such as a pen and a rectangular mass. A new methodology is proposed in order to solve the tire–road contact pressure. The sensor-array-embedded tires can provide more reliable and exact tire information to the vehicle than traditional indirect estimation methods. A rubber tire with a diameter of 80 mm was selected as the research object to use flexible pressure sensor arrays to detect the contact pressure of tires. Because of its excellent performance, the 1 wt% CNT doping spinosum pressure sensor using the abrasive paper template no. 600 was used to detect the contact pressure of the tire. In order to fabricate an intelligent tire, as shown in Figure 6d, a 2 × 3 pixel sensing array sandwiched between cross-arrays of copper electrodes was designed to detect the deformation of the tire. This 2 × 3 pixel sensing array was adhered to the tread of the tire using 3M tape. Then, the tire was installed on a test platform (Figure 6e) and compressed for ten cycles with a downward distance of 0.7 mm. Cross-locating technology was used to obtain the changes in the 6-pixel signal of the sensor array under the compression progress. Figure 6f shows the signal of 6 pixels. It can be seen that the signal changes of 5 and 6 pixels are most apparent, and the signal changes of 1–4 pixels are relatively small. From the experimental results, the pressure distribution at different positions of the tire can be detected in real time by coupling the sensor array into the tire, which is of great significance for the realization of intelligent tires.

## 4. Conclusions

In summary, we have proposed a sensitive capacitive pressure sensor with a broad detection range inspired by the skin epidermis. A simple and low-cost fabrication process was proposed for CNT/PDMS-based spinosum pressure sensors through using the abrasive paper templates. Notably, the spinosum microstructure and doping content of CNTs can effectively improve the performance of pressure sensors: high sensitivity (0.25 kPa^−1^), wider pressure range (~500 kPa), fast response time (20 ms) and excellent stability over 10,000 cycles. In addition, the effects of the mesh number of abrasive papers and CNT doping content on the sensing property are theoretically analyzed through simulations and experiments. Furthermore, a sensor array was manufactured for mapping the spatial distribution of pressure, which shows great potential for intelligent monitoring. Finally, a new methodology is proposed in order to solve the tire–road contact pressure and estimate related parameters by introducing a sensor array with a tire. Practically, this paper fabricated a bioinspired, cost-effective, broad-range, high-sensitivity, flexible sensor and opened a new patch to intelligently monitor the contact pressure of tires.

## Figures and Tables

**Table 1:** Comparison of the performance between our work and recently reported flexible capacitive pressure sensors.

Dielectric Layer	Materials	Sensing Range (kPa)	Sensitivity (kPa^−1^)	Response Time (ms)	Ref
pillars	SU8	0–30	0.0065	70	[41]
porous	PS/graphene/MWCNTs	0–4.5	0.062	45	[42]
microdome	PDMS	0.5–10	0.055	200	[43]
pyramid	PDMS	0.5–6	0.185	-	[44]
microridge	PDMS	0–10	0.148	20	[45]
spinosum	CNT/PDMS	0–500	0.25	20	This study

## Data Availability

The data presented in this study are available on request from the corresponding author.
